# Spatiotemporal Control of Intercellular Crosstalk: A New Therapeutic Paradigm for Halting Acute Kidney Injury to Chronic Kidney Disease Transition

**DOI:** 10.3390/biom16040559

**Published:** 2026-04-09

**Authors:** Hua Su, Kaixin Song

**Affiliations:** Department of Nephrology, Union Hospital, Tongji Medical College, Huazhong University of Science and Technology, Wuhan 430022, China; dr_suhua@hust.edu.cn

**Keywords:** AKI, CKD, cellular crosstalk, spatiotemporal regulation, therapeutic targeting

## Abstract

The transition from acute kidney injury (AKI) to chronic kidney disease (CKD) represents a dynamic and multistage pathological process driven by maladaptive intercellular communication. Rather than resulting from isolated cellular injury, AKI-CKD progression unfolds through a spatially and temporally coordinated dysregulation of cellular networks. In the acute phase, damaged tubular epithelial cells act as instigators, releasing damage-associated molecular patterns (DAMPs) and activating a storm of inflammatory crosstalk among immune cells, endothelium, and fibroblasts. During the subacute repair phase, imbalance in macrophage polarization (M1 persistence/M2 dysfunction) and the emergence of senescent tubular cells with a senescence-associated secretory phenotype (SASP) together create a pro-fibrotic microenvironment. In the chronic phase, activated myofibroblasts—derived from multiple sources—establish self-sustaining feedback loops via autocrine signaling, mechanical memory from the stiffened extracellular matrix (ECM), and ongoing dialogue with immune and resident cells, ultimately leading to irreversible fibrosis. Current therapeutic strategies focused on single molecular targets often fail to disrupt this resilient network homeostasis. Therefore, we propose a paradigm shift toward spatiotemporally precise network-remodeling therapies, which require integrated use of liquid biopsy-based staging, smart nanocarriers for cell-specific delivery, and AI-powered multi-omics modeling. This review systematically delineates the evolving cell-to-cell communication networks across AKI-CKD continuum and highlights innovative strategies to intercept disease progression by targeting the pathophysiology of cellular crosstalk.

## 1. Introduction

The transition from acute kidney injury (AKI) to chronic kidney disease (CKD) has emerged as a major clinical challenge in the field of nephrology worldwide, significantly exacerbating the global healthcare burden. Studies have shown that even mild or transient AKI episodes increase the risk of CKD by 2.36-fold, with higher AKI stages and longer durations correlating with a greater likelihood of progression to CKD or end-stage renal disease [1]. In patients with pre-existing CKD, AKI episodes further accelerate disease progression and are associated with a marked increase in all-cause mortality [2]. In clinical settings such as cardiac surgery and sepsis, the incidence of subsequent CKD in AKI patients can reach 24%, and this transition is often insidious, frequently lacking typical symptoms in the early stages [3]. Moreover, the AKI-to-CKD transition is closely linked to multisystem complications, including cardiovascular disease and bone metabolic abnormalities, which profoundly affect long-term patient outcomes [4].

Although the pathological significance of the AKI-to-CKD transition is widely recognized, current research predominantly focuses on molecular mechanisms of individual cell types—such as ferroptosis or mitochondrial dysfunction in tubular epithelial cells, or phenotypic switching in macrophages—while overlooking the central role of dynamic intercellular communication within the renal microenvironment. The kidney is a structurally complex organ that involves intricate interactions among various cell types during injury and repair, including tubular epithelial cells (TECs), macrophages, endothelial cells, and fibroblasts [5]. Existing studies often analyze pathological alterations in isolated cell populations in a reductionist manner, failing to elucidate how aberrant intercellular crosstalk across different stages drives maladaptive repair. For instance, the inflammatory signaling between macrophages and TECs, the interactions between endothelial cells and fibroblasts that promote fibrosis, and the dysregulation crosstalk among resident renal cells, immune cells, and fibroblasts are spatiotemporal dysregulation in intercellular communication that are critical drivers of AKI progression, yet systematic investigations in this area remain scarce [6,7,8]. This research gap has led to a lack of effective clinical strategies targeting aberrant intercellular communication, hindering efforts to fundamentally halt disease progression.

The AKI-to-CKD transition represents a progressive transformation from a “healthy cellular communication network” to a “pathological communication network in the kidney.” Under physiological conditions, the renal microenvironment maintains homeostatic communication among TECs, macrophages, endothelial cells, and other cell types through cytokines, exosomes, direct contact, and calcium signaling [9]. Following AKI, injury signals trigger a remodeling of intercellular communication patterns, with pro-inflammatory and pro-fibrotic signals becoming predominant, establishing a vicious cycle that drives disease progression from acute injury to chronic fibrosis [10]. Spatial transcriptomic studies have revealed that spatiotemporal dysregulation of intercellular communication persists throughout the disease course, and this sustained communication disorder is a key determinant of AKI chronicity [11]. Therefore, disrupting pathological intercellular communication and restoring a healthy network have become critical therapeutic goals, necessitating a spatiotemporally precise strategy that defines “when to intervene, which cellular interactions to target, and by what means” [12]. This review systematically dissects the mechanisms underlying pathological intercellular communication among key cell populations across different stages along the temporal axis of the AKI-to-CKD transition, and further explores spatiotemporally precise intervention strategies, aiming to provide new theoretical foundations and therapeutic insights for halting disease progression. In contrast to previous reviews that have largely focused on isolated molecular pathways or single-cell responses, this review innovatively integrates the spatiotemporal dynamics of multicellular interactions across the transition from AKI to CKD. We propose a strategic shift in research focus: from targeting single molecules toward reconstructing the complete cellular communication network.

## 2. Acute Phase of AKI: PTECs-Dominated “Crisis Initiation” and Burst of Inflammatory Intercellular Communication

The acute phase of AKI refers to the immediate stress response stage occurring within hours to days after renal injury, during which the kidney faces initial insults such as ischemia–reperfusion, sepsis, or nephrotoxic agents. The core pathological feature of this phase is the role of tubular cells, particularly proximal tubular epithelial cells (PTECs), as ‘crisis initiators’. Through the release of damage-associated molecular patterns (DAMPs), mitochondrial dysfunction mediated burst of reactive oxygen species (ROS), and programmed necrosis/pyroptosis, these cells emit potent injury signals to the innate immune system, rapidly activating a multicellular network of pathological communication and ultimately triggering an inflammatory storm and acute renal tissue damage [7,11].

### 2.1. PTECs: Injury and Signal Release as ‘Crisis Initiators’ in the Acute-Phase

PTECs are the most vulnerable cell type during the acute phase of AKI due to their high metabolic activity, abundant mitochondria, and the hyperosmolar environment within the renal tubules. Following injury, PTECs initiate the release of ‘crisis signals’ through multiple pathways, laying the foundation for pathological intercellular communication.

First, the extensive release of DAMPs is a central initiating event. Injuries such as ischemia, toxins, or proteinuria directly compromise the membrane integrity of PTECs, leading to the release of DAMPs into the renal interstitial microenvironment. These include high mobility group box 1 (HMGB1), adenosine triphosphate (ATP), heat shock proteins (HSPs), and uric acid [13,14]. A study by Tang et al. demonstrated that acute exposure to albumin induced time- and dose-dependent expression of IL-8 in human PTECs, a process dependent on NF-κB activation and associated with ROS generation. As a key chemokine, IL-8 further recruits immune cells [15]. HMGB1, a canonical DAMP, can be passively released from necrotic cells or actively secreted by stressed cells into the microenvironment, where its N-terminal domain binds to the receptor for advanced glycation end products (RAGE) on immune cells to initiate inflammatory signaling [16]. ATP amplifies inflammatory responses and cellular injury by activating purinergic receptors (e.g., P2X7R) on immune cells and PTECs [17]. Spatial transcriptomic studies have confirmed that within hours of AKI, elevated concentrations of HMGB1 and ATP are detected around injured PTECs, with distributions that closely overlap with areas of immune cell infiltration [11].

Second, mitochondrial dysfunction and the burst of ROS exacerbate injury signaling. PTECs are rich in mitochondria, and acute injury directly disrupts mitochondrial structure, leading to electron transport chain dysfunction and massive production of mitochondrial ROS (mtROS) [18,19]. Gao et al. found that in sepsis-induced AKI, PTECs exhibited mitochondrial swelling and loss of membrane potential. Excessive ROS generation directly damaged PTECs through lipid, protein, and DNA oxidation, while simultaneously activating the NF-κB pathway to promote the transcription of inflammatory cytokines [20]. Furthermore, mitochondrial DNA (mtDNA) released from damaged mitochondria itself serves as a critical DAMP, activating innate immunity in AKI [21].

Finally, programmed necrosis and pyroptosis amplify ‘crisis signals’. During the acute phase of AKI, PTECs primarily undergo necroptosis and pyroptosis, both of which are accompanied by the release of potent inflammatory signals [22,23]. Xu et al. demonstrated that in cisplatin-induced AKI, PTECs undergo necroptosis mediated by receptor-interacting protein kinase 1 (RIPK1), RIPK3, and mixed lineage kinase domain-like protein (MLKL). Activation of this pathway leads to membrane rupture and the release of large quantities of DAMPs and inflammatory cytokines [24]. Pyroptosis is mediated by gasdermin D (GSDMD) or gasdermin E (GSDME). Cisplatin stimulation in renal tissue induces GSDME mediated release of inflammatory factors and cell death [25].

### 2.2. Core Intercellular Communication Network in the Acute Phase: Inflammation-Driven Maladaptive Crosstalk

**Figure 1:**
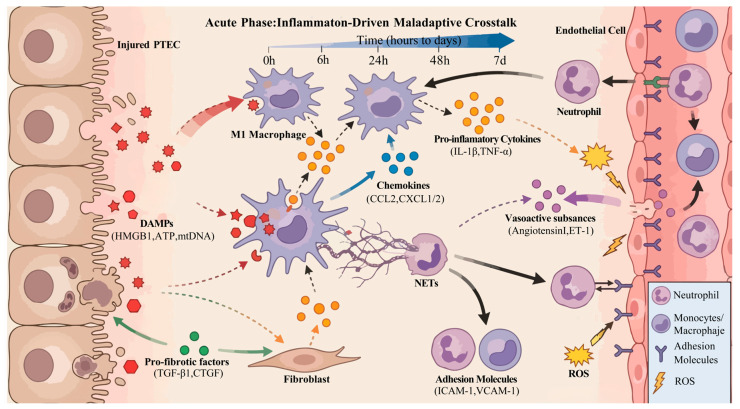
Inflammation-driven maladaptive cellular crosstalk during the acute phase of AKI.

Intercellular communication during the acute phase of AKI is centered on inflammatory amplification, involving multiple cell types including tubular cells, innate immune cells, endothelial cells, and fibroblasts. Through soluble mediators (DAMPs, inflammatory cytokines, chemokines) and cell surface molecule interactions, a rapidly activated pathological communication network is established (Figure 1).

#### 2.2.1. PTECs ↔ Immune Cells: Inflammation Initiation and Amplification

This is the most critical communication pathway in the acute phase. DAMPs released from injured PTECs, such as HMGB1, ATP, and mtDNA, act as chemotactic and activation signals, rapidly recruiting and activating resident renal interstitial macrophages as well as circulating neutrophils, monocytes, and dendritic cells [5,26,27,28].

Resident renal interstitial macrophages are among the first to sense DAMP signals, becoming activated through receptors such as RAGE, TLR9, and P2X7R. They rapidly polarize toward a pro-inflammatory M1 phenotype, secreting large amounts of chemokines (CCL2, CXCL1, CXCL2) and inflammatory cytokines (IL-1β, TNF-α, IL-6) [8,29]. A study by Cao et al. demonstrated that inhibition of NF-κB significantly reduced macrophage infiltration and MCP-1 (CCL2) expression after ischemia–reperfusion (I/R) injury, alleviating tubular necrosis [30]. Among these, CCL2 further recruits circulating monocytes into the kidney, where they differentiate into macrophages and expand the pro-inflammatory macrophage pool. In turn, IL-1β and TNF-α act in a paracrine manner on surviving PTECs, exacerbating their mitochondrial damage and DAMP release, thereby establishing a vicious cycle of “tubular injury–macrophage activation” [7,31].

DAMPs, along with CXCL1, CXCL2 secreted by macrophages, rapidly recruit neutrophils to the injury site. Neutrophils release myeloperoxidase (MPO), elastase, and neutrophil extracellular traps (NETs), directly compromising the integrity of tubular and endothelial cells while releasing additional inflammatory cytokines that amplify local inflammation [6,32]. NET release further damages tubules and accelerates NET formation, creating a vicious cycle [33]. Chujo et al. found that atrial natriuretic peptide (ANP) reduced neutrophil activation by inhibiting CINC-1 (CXCL1) and MPO expression, thereby attenuating I/R-induced tubular necrosis (Table 1) [34]. Studies have shown that renal neutrophil infiltration peaks at 48 after AKI, and blocking NET formation confers renoprotective effects [35].

#### 2.2.2. Endothelial Cells ↔ Immune Cells: “Vascular Gating” in Inflammatory Infiltration

Renal endothelial cells, which constitute the vascular barrier, regulate inflammatory cell infiltration during the acute phase through bidirectional communication with immune cells, acting as ‘vascular gatekeepers’. Following acute injury, ROS released from PTECs and TNF-α and IL-1β secreted by macrophages rapidly activate renal endothelial cells, leading to upregulation of adhesion molecules, including intercellular adhesion molecule-1 (ICAM-1) and vascular cell adhesion molecule-1 (VCAM-1) on their surface [9,36]. These adhesion molecules bind to integrins (LFA-1, VLA-4) on circulating neutrophils and monocytes, promoting immune cell adhesion to the vascular wall and subsequent transendothelial migration into the renal interstitium, thereby completing the recruitment and infiltration of inflammatory cells [37,38,39]. Activated endothelial cells also secrete chemokines such as CXCL8 and CCL5, further enhancing immune cell chemotaxis. In addition, endothelial cells can release molecules such as thromboxane A2 and angiotensin II, leading to renal vasoconstriction, exacerbating tissue hypoxia, and indirectly amplifying tubular injury [40,41]. Conversely, infiltrating immune cells can release inflammatory factors that further compromise endothelial cell integrity and disrupt vascular barrier function, creating a positive feedback loop of “endothelial activation–immune infiltration” [36]. Furthermore, NETs positively correlate with the severity of AKI; pharmacological inhibition of NET release or promotion of NET degradation in mice reduces renal microvascular endothelial cell injury and reverses renal pathological changes and creatinine levels [42].

#### 2.2.3. PTECs ↔ Fibroblasts: The “Early Warning” of Fibrosis

Although fibroblast activation is a core event in the chronic phase, communication between PTECs and fibroblasts during the acute phase sets the stage for subsequent fibrosis [43]. During the acute phase of AKI, injured PTECs release pro-fibrotic factors such as transforming growth factor-β1 (TGF-β1) and connective tissue growth factor (CTGF), pre-activating renal interstitial fibroblasts [44,45]. Moreover, studies have confirmed that within 48 h after AKI, renal interstitial fibroblasts are extensively activated, and these activated fibroblasts exhibit increased secretion of inflammatory cytokines, further promoting tubular cell injury [46]. Fibroblast-specific knockout of PU.1/Spi1 reduces interstitial inflammatory cytokine levels, attenuates tubular cell apoptosis, and ameliorates AKI [46]. Additionally, fibroblast-specific deletion of pyruvate kinase M2 (PKM2) disrupts hepatocyte growth factor (HGF) signaling, promotes tubular cell death, and exacerbates AKI [47]. These findings suggest that tubular-fibroblast communication during the acute phase is as an early driver of the AKI-to-CKD transition. However, some studies have also shown that activation of mTORC1 and mTORC2 signaling in fibroblasts during the acute phase may help protect against tubular cell death and mitigate renal injury [48].

#### 2.2.4. Immune Cell ↔ Immune Cell Crosstalk: Cascade Amplification of Inflammatory Signals

Intercommunication among immune cells, including macrophages, neutrophils, and monocytes, is critical for the formation of the inflammatory storm during the acute phase. After AKI, macrophages arrive at the injury site first and enhance neutrophil recruitment, thereby amplifying the inflammatory response [49]. Lactylation of HMGB1 in macrophages triggers the release of mitochondrial DNA from neutrophils, promoting NETs formation [50]; macrophages have also been shown to mediate neutrophil recruitment and renal injury in Shiga toxin-induced hemolytic uremic syndrome [51]. Conversely, inhibiting NET formation promotes the infiltration and survival of growth arrest-specific protein 6 (GAS6)-expressing macrophages, conferring renoprotection [35]. In acute rejection following kidney transplantation, neutrophil depletion suppresses natural killer (NK) cell activation and reduces macrophage infiltration but increases myeloperoxidase (MPO)-producing monocytes [52]. This ‘cross-activation’ among immune cells rapidly amplifies inflammatory signals, leading to widespread renal tissue damage while establishing the foundation for subsequent chronic inflammation.

#### 2.2.5. Supplementary Pathway: PTECs ↔ Endothelial Cells

Beyond the core networks described above, direct communication between PTECs and endothelial cells during the acute phase should not be overlooked. DAMPs may activate both renal vascular endothelial cells and PTECs, promoting the upregulation of adhesion molecules and the release of additional pro-inflammatory mediators and ROS, which in turn facilitate platelet activation and aggregation, leading to microvascular dysfunction, hypoxia, and tissue injury [53]. During AKI, pro-inflammatory cytokines produced by injured PTECs act on endothelial cells increasing their permeability [54]. Conversely, endothelin-1 (ET-1) released from activated endothelial cells can act back on TECs, exacerbating renal injury after AKI [55]. This bidirectional communication disrupts the homeostasis of the renal microcirculation and the tubule-vascular interface, further amplifying acute injury.

### 2.3. Pathological Significance and Outcomes of Intercellular Communication in the Acute Phase

The intercellular communication network during the acute phase of AKI is centered on rapid inflammatory activation (Figure 1). Persistent pathological intercellular communication during this phase induces remodeling of the renal microenvironment, driving macrophages, fibroblasts, and other cells into a persistently activated state, thereby laying the subsequent maladaptive repair in the subacute phase and fibrosis in the chronic phase. Therefore, targeting key nodes of intercellular communication during the acute phase—such as DAMPs release or NF-κB activation—represents a critical intervention window for halting AKI progression and the AKI-to-CKD transition. Existing studies have demonstrated that neutralizing the DAMP molecule HMGB1 with specific antibodies or blocking its receptor binding significantly inhibits intrarenal inflammatory cascades in models of sepsis and I/R injury, reducing tubular cell death and interstitial fibrosis [17,56,57]. Moreover, inhibition of NF-κB (e.g., using the inhibitor BAY 11-7082 or 6-gingerol) has been shown to effectively improve renal function and delay progression to CKD [58,59].

Injured PTECs release DAMPs (HMGB1, ATP, mtDNA), which activate interstitial macrophages towards an inflammatory M1 phenotype. Activated M1 macrophages secrete pro-inflammatory cytokines (IL-1β, TNF-α) that perpetuate tubular damage and activate endothelial cells. They also release chemokines (CCL2, CXCL1/2) that recruit circulating immune cells. Activated endothelial cells upregulate adhesion molecules (ICAM-1/VCAM-1), facilitating the extravasation of neutrophils and monocytes. Infiltrating neutrophils release NETs and Reactive ROS, amplifying tissue injury. Endothelial cells also release vasoactive substances (Angiotensin II, ET-1). Simultaneously, injured PTECs secrete pro-fibrotic factors (TGF-β1, CTGF), leading to the early activation of pro-inflammatory fibroblasts. Solid arrows indicate cell migration; dashed arrows indicate the secretion and action of soluble factors; lightning symbols indicate injury by ROS. PTECs, Proximal Tubular Epithelial Cells; DAMPs, Damage-Associated Molecular Patterns; HMGB1, high mobility group box 1; ATP, adenosine triphosphate; mtDNA, mitochondrial DNA; ICAM-1, intercellular adhesion molecule-1; VCAM-1, vascular cell adhesion molecule-1; NETs, Neutrophil Extracellular Traps; ROS, Reactive Oxygen Species; ET-1, endothelin-1; TGF-β1, transforming growth factor-β1; CTGF, connective tissue growth factor; ROS, reactive oxygen species.

## 3. Subacute/Repair Phase of AKI: Microenvironment Remodeling Driven by Immune Polarization Imbalance and Senescent Cell “Noise”

The subacute or repair phase of AKI typically occurs from days to weeks after the initial injury and represents a critical turning point that determines whether the kidney undergoes successful repair or progresses to CKD. The core pathological features of this phase are the dual effects of immune cell functional remodeling (centered on macrophage polarization imbalance) and the persistent tubular cell senescence with its associated senescence-associated secretory phenotype (SASP). The former results in insufficient repair signaling, while the latter acts as a new chronic inflammatory source. Together, through complex intercellular communication network, they shape a maladaptive repair microenvironment that ultimately drives the progression of renal tissue towards fibrosis [60,61,62].

### 3.1. Macrophage Polarization Imbalance—A Pathological Shift from Damage Clearance to Repair Arrest

Macrophages are central regulators of the renal interstitial microenvironment during the subacute/repair phase of AKI, and their function depends on dynamic polarization. Under normal repair conditions, pro-inflammatory M1 macrophages first clear necrotic tissue, after reparative M2 macrophages take the lead in regeneration. However, when the polarization process becomes dysregulated, the repair program stalls and fibrosis is initiated [63].

#### 3.1.1. Macrophage Polarization in Normal Repair: Dynamic M1 → M2 Coordination

In the early subacute phase following AKI, DAMPs, such as HMGB1 and ATP released upon initial injury (e.g., I/R, sepsis) recruit circulating monocytes and drive their differentiation into M1 phenotype [64,65]. M1 macrophages through the secretion of TNF-α, IL-1β, and ROS, on one hand exacerbate apoptosis and necrosis of injured TECs, and on the other hand clearing the way for subsequent repair by phagocytosing necrotic debris and degrading casts [66,67,68]. As the repair process advances into its middle phase, the accumulation of anti-inflammatory factors in the renal interstitial microenvironment triggers the phenotypic switch of macrophage from M1 to the M2 phenotype [63].

#### 3.1.2. Two Core Patterns of Polarization Imbalance: M1 Persistence and M2 Functional Deficiency

M1 macrophage persistence: This refers to the failure of pro-inflammatory macrophages to subside in a timely manner, resulting in their sustained infiltration and representing a core mechanism driving maladaptive repair. Studies have shown that infiltrating monocyte-derived macrophages, a core M1 subset, are associated with chronic inflammation and fibrosis, and communicate with fibroblasts though insulin-like growth factor (IGF) signaling [69]. NF-κB can directly bind to the HIF-1α promoter to enhance its transcription, mediating glycolytic metabolic reprogramming in macrophages, maintaining the pro-inflammatory phenotype, and promoting the transition from AKI to CKD [70]. Intervention with C-176 reduces the M1 macrophage proportion and attenuates inflammation, thereby mitigating AKI and improving renal function [71].

M2 macrophage functional deficiency: This manifests as either an insufficient number of reparative macrophages or impaired functionality, preventing the effective initiation of tissue repair programs. Previous studies have shown that Periostin promotes M2 macrophage proliferation and the secretion of repair factors through integrin-β1 signaling; functional deficiency of this pathway renders M2 macrophages unable to exert their pro-reparative effects, exacerbating renal injury [72]. Aquaporin 1 (AQP1) mediates M2 polarization via the PI3K pathway; insufficient activation of this pathway leads to reduced secretion of anti-inflammatory factors by M2 macrophages, thereby failing to improve the inflammatory microenvironment [73]. M2 macrophages promote the secretion of IL-10 and inhibit the release of TNF-α;. functional deficiency of M2 macrophages results in persistent inflammation [74]. Furthermore, M2 macrophages derived from peritoneal dialysis fluid have been shown to promote TECs proliferation, reduce inflammation, and ameliorate renal injury [66].

### 3.2. TECs Senescence: From Acute Stress Protection to Chronic Stagnation Exacerbating Inflammation and Fibrosis Initiation

Following AKI, the repair response of TECs is critical in determining whether renal function recovers or progresses to CKD [75]. During the acute stress phase of AKI, TECs senescence serves as an adaptive and protective mechanism; stress-induced senescent TECs resist necrosis and apoptosis, thereby promoting the proliferation and regeneration of healthy TECs [76]. Studies have shown that after AKI, p21 knockout mice are more susceptible to ischemia-induced acute renal failure, and the transient cell cycle arrest mediated by the p21 pathway is essential for TECs proliferation following I/R injury [77,78].

However, upon entering the subacute/repair phase, persistent tubular senescence shifts from protection to chronic stagnation, characterized by loss of proliferative capacity, cell cycle arrest, and an inflammatory secretory phenotype, ultimately leading to tubular atrophy and irreversible renal dysfunction [79]. Sustained G2/M phase arrest in TECs is typically accompanied by the development of a SASP, exacerbating maladaptive repair and driving the progression from AKI to CKD [80,81]. Research corroborates that in injured kidneys, inflammatory TECs promote the secretion of fibrosis- and senescence-associated molecules [82]. IL-1β triggers G2/M cell cycle arrest and cellular senescence under hypoxic renal injury conditions [83]. Additionally, senescent TECs exhibit downregulation of PGC-1α expression, accumulation of mitochondrial DNA damage, and insufficient ATP production, further exacerbating the loss of cellular functional [84,85]. Annexin A1 (ANX1) mediates mitochondrial calcium overload to generate pro-senescence signals, driving the progression from AKI to CKD [86].

### 3.3. Core Intercellular Communication Network: Multicellular Interactions Driving Maladaptive Repair

**Figure 2:**
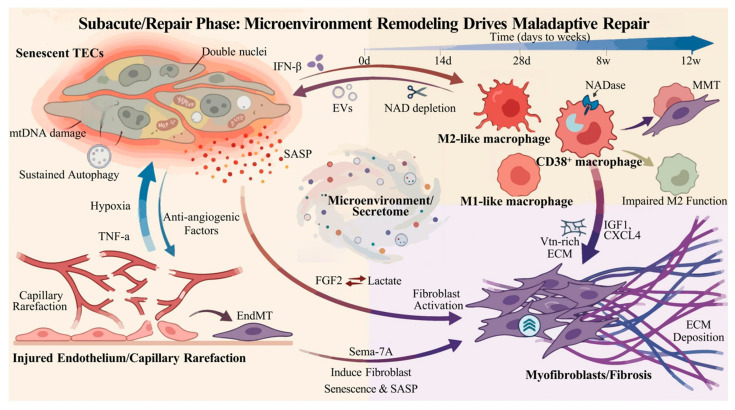
The maladaptive repair cycle driven by microenvironment remodeling during the subacute phase of kidney injury.

Intercellular communication during the subacute/repair phase centers on inflammatory amplification-repair inhibition-fibrosis initiation, involving multiple cell types including TECs, macrophages, fibroblasts, and endothelial cells. A network of malignant interactions is formed through soluble mediators (cytokines, chemokines), extracellular vesicles (e.g., exosomes), and metabolites (e.g., lactate, succinate) (Figure 2) [87,88,89].

#### 3.3.1. Senescent TECs ↔ Macrophages: Self-Reinforcing Inflammation

Single-nucleus RNA sequencing (snRNA-seq) results have revealed abnormally active intercellular communication between maladaptively repaired TECs and macrophages after AKI [90]. Following injury, a subset of TECs exhibit characteristics of cellular senescence. Extracellular vesicles derived from these senescent cells induce M2 polarization of macrophages and promote TGF-β secretion through non-coding RNAs, thereby facilitating the progression from AKI to CKD [91]. Conversely, Cd38hi macrophages deplete renal NAD through CD38 NADase activity, exacerbating TECs senescence and serving as a key profibrotic cell type in the AKI-to-CKD transition [92]. Furthermore, following rhabdomyolysis-induced AKI, activated macrophages promote TECs senescence through the Vav1/Rac2/NF-κB signaling pathway [93]. Additionally, macrophage-derived IFN-β binds to the type I interferon receptor on TECs, inducing polyploidization, which is closely associated with maladaptive repair after AKI [94].

#### 3.3.2. Macrophages → Fibroblasts: Initiation Signals for Fibrosis

Macrophages serve as a critical bridge connecting inflammation and fibrosis. Integrated single-cell RNA sequencing and spatial transcriptomic analyses have revealed that extracellular matrix remodeling-associated macrophages communicate with fibroblasts through insulin-like growth factor (IGF) signaling, promoting AKI progression [69]. Macrophage-derived vitronectin (Vtn)-rich extracellular matrix scaffolds activate fibroblasts by stimulating integrin αvβ5 and Src kinase signaling, driving the transition from AKI to CKD [95]. Moreover, SPP1-positive macrophages drive myofibroblasts activation after renal injury in a CXCL4-dependent manner [96]. In addition, macrophage-to-myofibroblast transition (MMT) is an important contributor to renal fibrosis [97]. Studies have shown that the HIF-1α/A2BAR signaling pathway alleviates renal fibrosis after I/R injury by preventing MMT [98].

#### 3.3.3. TECs ↔ Endothelial Cells: Microcirculation Dysfunction and Impaired Repair

Maladaptively repaired TECs following AKI disrupt peritubular capillary endothelial cells through secreted factors, leading to capillary rarefaction. Conversely, local hypoxia caused by microvascular pathology impedes tubular repair [87]. Studies have reported that VEGF-A-rich extracellular vesicles secreted by TECs after AKI promote peritubular capillary repair and slow AKI progression [99]. Furthermore, when co-cultured with endothelial cells, TECs can sense endothelial-derived TNF-α and produce inflammatory mediators [100].

#### 3.3.4. TECs/Endothelial Cells ↔ Fibroblasts: The Fibrotic ‘Amplification Circuit’

Following AKI, TECs undergoing maladaptive repair exhibit prolonged G2/M phase cell cycle arrest and develop a SASP. The cytokines they secrete act on fibroblast in a paracrine manner, accelerating extracellular matrix production [101]. Moreover, persistent autophagy after AKI activates early growth response 1 (EGR1) through the MAPK/ERK pathway, inducing the expression and secretion of fibroblast growth factor 2 (FGF2) by TECs, which in turn activates fibroblasts and promotes renal fibrosis [102,103]. Additionally, during AKI progression, lactate produced by injured TECs is taken up by interstitial fibroblasts, inducing their activation and proliferation [104]. Lactate also acts as a signaling mediator, upregulating HGF following fibroblast activation and amplifying profibrotic signals (Table 1) [105].

The endothelial-to-mesenchymal transition (EndoMT) after AKI is an important cause of vascular rarefaction, driving the progression from AKI to CKD [106]. Following vascular rarefaction, chronic hypoxia develops in renal tissue, activating TGF-β and extracellular matrix molecular regulation, which gradually leads to renal interstitial fibrosis [107]. Single-cell sequencing results have shown that PHD inactivation in endothelial cells promotes EndoMT, leading to poor renal repair after AKI [108]. Furthermore, Semaphorin-7A (Sema-7A) secreted by aberrant endothelial cells induces premature senescence in fibroblasts, which become a source of SASP that in turn affects surrounding healthy TECs and endothelial cells, establishing a vicious cycle [109].

### 3.4. Pathological Significance and Outcomes of Intercellular Communication in the Subacute/Repair Phase

During the subacute/repair phase, macrophage polarization imbalance (M1 persistence and M2 functional deficiency), persistent SASP, and metabolic communication dysregulation collectively lead to the gradual formation of a pro-fibrotic microenvironment in the renal interstitium, characterized by sustained elevation of inflammatory factors, fibroblast activation, microvascular rarefaction, and abnormal accumulation of metabolites, ultimately driving the progression from AKI to CKD (Figure 2). Therefore, targeting key nodes in this phase has become a central strategy for blocking the AKI-to-CKD transition. Studies have shown that mesenchymal stem cell-derived extracellular vesicles (MSC-EVs) promote the polarization of CX3CR1^+^ macrophages toward the M2 phenotype through the TXNIP-IKKα/NF-κB pathway, contributing to renal repair after AKI [110]. Concurrently, MSC-EVs can specifically reduce tubular atrophy and renal interstitial fibrosis during the AKI-to-CKD transition by regulating the Ras-pERK-Ets1-p53 signaling pathway. Moreover, combined treatment with human umbilical cord MSC-EVs (HUMSC-EVs) and senolytic drugs (dasatinib and quercetin) exerts enhanced renoprotective effects after AKI [111]. Application of the glycoside opine inhibits MCT4, a key molecule for lactate transport, thereby suppressing the activation of pro-inflammatory endothelial cells and consequently inhibiting AKI progression [106]. Additionally, administration of an endothelin A antagonist improves both macrovascular and microvascular function and prevents the transition from AKI to CKD [112,113].

The complex multicellular crosstalk converging on a dysregulated microenvironment/secretome, which leads to failed repair and fibrosis. Injured TECs adopt a senescent phenotype, characterized by cellular enlargement, polyploidization (double nuclei), mtDNA damage, and sustained autophagy. These cells release a SASP. Dysregulated macrophages contribute to inflammation and fibrosis through persistent M1-like states, impaired M2 function, and MMT. Notably, CD38+ macrophages express NADase, depleting NAD+ and aggravating TEC senescence. Bidirectional signaling involving EVs and IFN-β occurs between TECs and macrophages. The injured endothelium undergoes capillary rarefaction and EndMT. A vicious cycle involving hypoxia, TNF-α, and anti-angiogenic factors is established between endothelial cells and TECs. Pro-fibrotic signals from senescent TECs (IL-1β, TGF-β, FGF2, Lactate), macrophages (IGF-1, CXCL4, Vtn-rich ECM scaffold), and injured endothelium (Sema-7A) converge to drive myofibroblast activation, proliferation, and excessive ECM deposition, ultimately resulting in kidney fibrosis. TECs, Tubular Epithelial Cells; mtDNA, mitochondrial DNA; SASP, senescence-associated secretory phenotype; MMT, Macrophage-to-Myofibroblast Transition; EVs, extracellular vesicles; EndMT, Endothelial-to-Mesenchymal Transition; ECM, extracellular matrix.

## 4. Chronic/Fibrotic Phase: Myofibroblast-Dominated Pathological Homeostasis and Multicellular Interaction Networks

Months to years after AKI onset, kidney injury enters a chronic progressive stage. The core pathological features of this phase are the activation and persistence of myofibroblasts, which drive irreversible fibrosis progression through the formation of self-sustaining feedback loops, ultimately leading to permanent loss of renal function and completing the pathological transition from AKI to CKD [114,115]. Within the renal tissue microenvironment, myofibroblasts form complex intercellular communication networks with immune cells, TECs, endothelial cells, and the extracellular matrix (ECM), collectively maintaining fibrotic pathological homeostasis. The molecular mechanisms and cellular interaction patterns underlying these networks have become critical targets for elucidating disease progression [116].

### 4.1. Core Driver: Myofibroblasts and Self-Sustaining Fibrotic Feedback Loops

In the chronically injured kidney, myofibroblasts replace inflammatory cells as the core effector cells driving pathological progression, and their origins are highly heterogeneous. In addition to conventional fibroblast-to-myofibroblast transition (FMT), epithelial–mesenchymal transition (EMT), and EndoMT, pericyte-to-myofibroblast transition (PMT) continuously replenishes the effector cell pool. Furthermore, the MMT represents a critical supplementary pathway during the chronic phase [117,118]. These cells acquire a contractile phenotype through expression of α-smooth muscle actin (α-SMA) and simultaneously initiate the synthesis and secretion of large quantities of ECM components, including collagen I and III, leading to excessive ECM deposition in the renal interstitium.

More critically, activated myofibroblasts maintain the stability of their fibrotic phenotype through self-sustaining feedback loops: Autocrine Activation: Secreted TGF-β1 activates myofibroblasts in an autocrine manner, reinforcing α-SMA and collagen expression through the Smad2/3 pathway [119]. Secreted FGF2 also promotes fibroblast proliferation and activation [120]. Inflammatory Amplification: Inflammatory cytokines within the fibrotic microenvironment stimulate fibroblast differentiation into myofibroblasts, and activated myofibroblasts secrete pro-fibrotic factors that sustain the local chronic inflammatory microenvironment, further promoting their own activation and proliferation [121]. Hypoxia Induction: Excessive ECM deposition leads to tissue hypoxia, which promotes renal parenchymal cell apoptosis and MMT through HIF-α, exacerbating the vicious cycle of fibrosis [122,123].

### 4.2. Intercellular Crosstalk: Multidimensional Regulation of the Fibrotic Network

**Figure 3:**
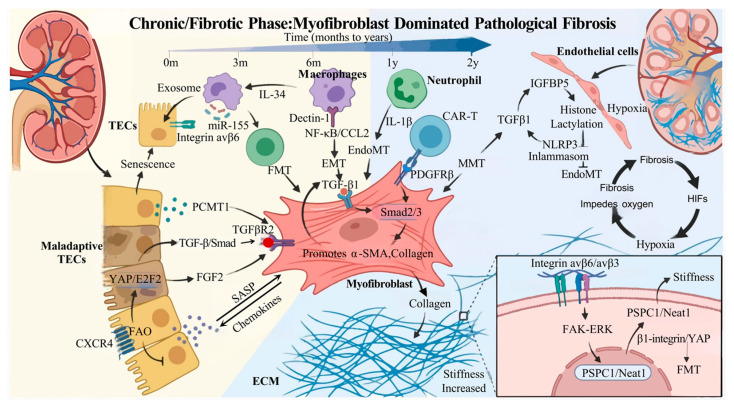
The multicellular interaction network and pathological homeostasis in chronic kidney fibrosis.

Fibrosis progression in the chronically injured kidney dose not result from the action of myofibroblasts alone; rather, it involves precise and sustained communication among multiple cell types that amplifies pathological signals. Multiple cellular interaction patterns constitute the core regulatory nodes of the fibrotic network (Figure 3).

#### 4.2.1. Fibroblast ↔ Immune Cell: Synergistic Reinforcement of Inflammation and Fibrosis

Bidirectional communication between fibroblasts and immune cells serves as a critical link sustaining chronic inflammation and fibrosis. Studies have shown that M2 macrophages, characterized by CD206^+^/CD163^+^ markers, are present at injury sites and are closely associated with the degree of renal fibrosis [124]. Knockdown of interferon regulatory factor 7 (IRF7) in macrophages inhibits MMT through cathepsin S (CTSS), suppressing sustained ECM production and ultimately attenuating renal fibrosis [125]. Dectin-1 expression is elevated in renal macrophages from CKD patients. Dectin-1 promotes macrophage infiltration through the Syk/NF-κB/CCL2-CCR2 axis while activates the TGF-β/Smad pathway to promote MMT. Genetic knockout of Dectin-1 or inhibition with laminarin significantly reduces renal fibrosis [126]. Chimeric antigen receptor T (CAR-T) cells specifically recognize platelet-derived growth factor receptor β (PDGFRβ) on the surface of fibroblasts, selectively inhibiting fibroblast activation, proliferation, and ECM synthesis, thereby significantly attenuating renal and multi-organ (cardiac, perivascular) fibrosis. This interaction has been validated in multiple CKD mouse models without observed toxicity, providing a novel approach for targeted fibrosis therapy [127]. Additionally, extensive neutrophil infiltrating occurs in the late stages of renal fibrosis, and these neutrophils activate fibroblasts by secreting IL-1β and other cytokines, thereby promoting fibrosis [128]. Conversely, activated fibroblasts increase secretion of chemotactic cytokines, which in turn promote macrophage migration and activation, further exacerbating renal inflammation and accelerating fibrosis progression [129].

#### 4.2.2. Fibroblast ↔ TECs: Sustained Driving of Maladaptive Repair

Maladaptive repair of TECs following AKI is a key initiating event triggering fibrosis, and aberrant communication between these cells and fibroblasts is critical for maintaining chronic injury. During the chronic phase, injured TECs fail to undergo normal repair and instead promote FMT and ECM deposition by secreting protein-L-isoaspartate O-methyltransferase 1 (PCMT1), which activates the TGF-β1/SMAD pathway through binding to TGF-β receptor type II (TGFBR2) [130]. Yes-associated protein (YAP) in TECs forms a complex with E2F2, promoting the secretion of FGF2, which activates renal interstitial fibroblasts in a paracrine manner [129]. Under high-glucose or injurious stimulation, TECs upregulate C-X-C chemokine receptor type 4 (CXCR4), which inhibits fatty acid oxidation by activating the β-catenin pathway, induces cellular senescence and promotes secretion of SASP factors, thereby facilitating fibroblast activation and ECM accumulation [131]. Activated fibroblasts, in turn, further secrete profibrotic ECM components and chemokines, establishing a vicious cycle of renal fibrosis [129,132].

#### 4.2.3. Fibroblast ↔ Endothelial Cell: A Vicious Cycle of Vascular Injury and Fibrosis

The interplay between injured endothelial cells and activated fibroblasts further exacerbates the pathological process during the chronic phase. Persistent chronic inflammation following AKI leads to endothelial cell senescence and capillary rarefaction. Hypoxia activates fibroblasts, contributing to renal fibrosis, while fibrosis impedes effective oxygen diffusion into the interstitium, in turn exacerbating hypoxia and perpetuating a vicious fibrotic cycle [132]. Insulin-like growth factor-binding protein 5 (IGFBP5) in endothelial cells promotes EndoMT and renal fibrosis mediated by NLRP3 through regulation of glycolysis-mediated histone lactylation [133]. Administration of the monoterpene glycoside paeoniflorin blocks endothelial cell-derived TGF-β1 paracrine signaling, reducing fibroblast activation and ECM deposition [134].

#### 4.2.4. TECs ↔ Immune Cells: Perpetuating the Chronic Inflammatory Milieu

Overexpression of integrin αvβ6 in TECs activates macrophages by promoting interleukin-34 (IL-34) secretion. Knockout of αvβ6 significantly attenuates not only renal fibrosis but also inflammation, particularly infiltration of pro-inflammatory macrophages [135]. Macrophage derived exosomes promote telomeric fragility and senescence in TECs through delivery of miR-155 [136]. Additionally, T cells stimulate TECs to produce chemokines by releasing pro-inflammatory factors, thereby recruiting additional T cells and other immune cells into the kidney and inducing the EMT [137].

#### 4.2.5. ECM Physical Signals: Mechanical Memory Regulation of Fibrosis

Alterations in the physical properties of the ECM represent a critical regulatory factor that cannot be overlooked during, establishing a mechanical memory of fibrosis through mechanosignaling–cellular response pathways. As ECM deposition persists, renal interstitial tissue stiffness increases significantly. This physical signal regulates PSPC1 and Neat1, triggering TGF-β1-induced transdifferentiation of renal progenitor cells into myofibroblasts through the β1-integrin–YAP pathway [138]. Inhibition of YAP/TAZ signaling in fibroblasts effectively blocks TGF-β1-induced FMT and ECM production [139]. Beyond matrix stiffness, ntegrin–focal adhesion signaling-mediated mechanotransduction also promotes renal fibrosis. Integrins αvβ6 and αvβ3 on fibroblast surfaces bind to collagen and fibronectin within the ECM, forming focal adhesion complexes that activate the FAK-ERK pathway, enhancing fibroblast proliferation and ECM synthetic capacity (Table 1) [140].

### 4.3. Pathological Significance and Outcomes of Intercellular Communication in the Chronic Phase

The chronic phase of the AKI-to-CKD transition is a pathological process dominated by myofibroblasts and characterized by synergistic multicellular interactions. The formation of a myofibroblast-dominated fibrotic network establishes an irreversible pathological homeostasis, ultimately leading to permanent loss of renal function (Figure 3). If the self-sustaining feedback loop of myofibroblasts remains continuously activated, multicellular interaction networks undergo aberrant amplification, and ECM physical signals persist, irreversible fibrotic changes in the kidney ensue, ultimately culminating in end-stage renal disease. Therefore, targeting key nodes in this phase may provide core strategies for chronic phase therapy. For example, verteporfin (VP) reduces myofibroblast activation and proliferation in the kidneys by modulating the phosphorylation of Smad2 and Smad3, attenuating renal inflammation and ECM deposition [141]. SIS3 ameliorates myofibroblast activation and reduces fibrosis and inflammation by inhibiting the TGF-β/Smad3 pathway [142]. The STAT6 inhibitor AS1517499 protects renal function by inhibiting fibroblast activation, reducing M2 macrophage polarization, and decreasing ECM protein production [143]. Additionally, melatonin improves mitochondrial function in CKD-derived MSCs through the PrP(C)-PINK1 pathway, inhibits cellular senescence, and enhances their reparative capacity, offering a new direction for cell-based therapies.

The chronic phase characterized by a myofibroblast dominated fibrotic microenvironment. The activated myofibroblast serves as the core effector, originating from resident fibroblasts (FMT), epithelial cells (EMT), endothelial cells (EndoMT), and macrophages (MMT). It sustains a contractile phenotype and drives ECM accumulation through self-reinforcing autocrine loops involving TGF-β1/Smad2/3 and FGF2. Injured TECs undergo maladaptive repair. They secrete PCMT1 to activate fibroblast TGFBR2 and release FGF2 driven by nuclear YAP/E2F2 complexes. Metabolic reprogramming, indicated by CXCR4 mediated inhibition of FAO, induces a SASP that further activates fibroblasts. Macrophage Dectin-1 signaling promotes direct MMT. A reciprocal loop exists where TECs-derived IL-34 recruits macrophages, while macrophage-derived exosomal miR-155 induces TECs senescence. CAR-T cells targeting PDGFRβ are shown as a potential therapeutic intervention. Capillary rarefaction leads to tissue hypoxia, activating HIFs. Endothelial IGFBP5 promotes histone lactylation and NLRP3 inflammasome activation, driving EndoMT. A vicious cycle is established where fibrosis physically impedes oxygen diffusion, exacerbating hypoxia. Increased ECM stiffness acts as a physical signal. Fibroblasts sense matrix rigidity via Integrins, activating the FAK-ERK pathway and upregulating PSPC1/Neat1 to trigger YAP signaling, thereby reinforcing the fibrotic phenotype. FMT, fibroblast-to-myofibroblast transition; EMT, epithelial–mesenchymal transition; EndoMT, endothelial-to-mesenchymal transition; MMT, macrophage-to-myofibroblast transition; ECM, extracellular matrix; TECs, tubular epithelial cells; FAO, fatty acid oxidation; SASP, senescence-associated secretory phenotype; CAR-T, chimeric antigen receptor T.

## 5. Clinical Relevance and the Limits of Current Biomarkers

AKI to CKD transition often occurs insidiously, making timely intervention difficult. Traditional renal function markers, such as serum creatinine, remain the cornerstone of clinical assessment; however, they exhibit significant limitations. Serum creatinine primarily reflects glomerular filtration function rather than tubular injury or interstitial fibrosis, and its levels are influenced by many factors including age, muscle mass, and hydration status. Moreover, substantial nephron loss may have already occurred before creatinine levels rise, rendering it a late marker of irreversible injury [105]. Therefore, there is an urgent need for more sensitive and specific biomarkers capable of capturing the dynamic pathological processes at different stages of the disease.

### 5.1. Urinary and Plasma Protein Biomarkers

Plasma IL-6 levels can predict the occurrence of AKI [144]. A multiplex biomarker panel combining neutrophil gelatinase-associated lipocalin (NGAL), kidney injury molecule-1 (KIM-1), liver-type fatty acid-binding protein (L-FABP), cystatin C, and HMGB1 demonstrates superior performance in predicting the AKI-to-CKD transition compared to single markers [145].

### 5.2. Exosome and miRNA Signatures

Urinary miR-494 levels are increased by 60-fold in patients with AKI, and its diagnostic detection precedes that of traditional creatinine [146]. Furthermore, miR-141 levels at the time of AKI onset correlate with non-recovery of renal function at 90 days [147]. Urinary miR-556-3p can predict renal recovery at 28 days following AKI [148]. miR-21 is the most extensively studied miRNA in this field, with studies demonstrating elevated urinary miR-21 levels in AKI patients [149], while in CKD patients, miR-21 is persistently upregulated in both urine and plasma, showing a negative correlation with estimated glomerular filtration rate (eGFR) and a positive correlation with the degree of interstitial fibrosis [150,151]. These findings underscore the potential of miRNA-based diagnostics for risk stratification and longitudinal monitoring.

### 5.3. Senescence-Associated Markers

Cellular senescence is a key driver of maladaptive repair following AKI. Recent studies have shown that a urinary clusterin-to-creatinine ratio (uCCR) > 124.5 μg/mmol is closely associated with P21^+^Ki67^−^ epithelial cell senescence and predicts adverse renal outcomes in CKD patients at three years [152].

### 5.4. Endothelial Dysfunction Markers

Endothelial dysfunction serves as both a contributor to and a consequence of AKI-to-CKD progression. Endothelial-associated biomarkers, including VCAM-1, AGPT2, and syndecan-1, can predict the occurrence of AKI [153]. Elevated plasma levels of ICAM-1, VCAM-1, FVIII, and E-selectin are strongly associated with CKD development and a ≥30% decline in eGFR [154].

In summary, although serum creatinine remains indispensable in routine clinical practice, its limitations in detecting subclinical disease progression underscore the necessity of adopting a multi-marker approach. Integrating urinary protein panels, exosomal miRNA signatures, senescence-associated molecules, and endothelial dysfunction markers provides a more comprehensive framework for staging, risk prediction, and therapeutic guidance during AKI-to-CKD progression. Future efforts should focus on validating these candidate biomarkers in large prospective cohorts and establishing clinically actionable thresholds.

## 6. Translational Perspectives: A Clinical Framework for Network-Targeted Therapy

### 6.1. Clinical Translational Pathways for Stage-Specific Intervention Strategies

Based on the three pathological phases of the AKI-to-CKD transition, we propose the following intervention strategies amenable to clinical trial translation:

Acute Phase: Targeting DAMP receptors or NF-κB inhibitors aims to halt the initial inflammatory storm. Preclinical studies have demonstrated that anti-HMGB1 antibodies or NF-κB inhibitors significantly attenuate AKI and delay CKD progression [56,57,58,59]. We recommend designing randomized controlled trials with intervention initiation within seven days after AKI onset, using the 90-day renal recovery rate and changes in renal fibrosis markers as primary endpoints.

Subacute/Repair Phase: Targeting senescent cells or modulating macrophage polarization. Recent clinical trials have preliminarily validated the safety of dasatinib plus quercetin in patients with diabetic kidney disease, along with improvements in senescence markers. We recommend conducting multicenter phase II trials using changes in urinary SASP factors and eGFR slope as surrogate endpoints.

Chronic/Fibrotic Phase: Targeting fibroblast activation or ECM mechanical signaling pathways aims to reverse established fibrosis. Recent studies have demonstrated for the first time that CAR-T cells can effectively eliminate fibroblasts in renal fibrosis [127], providing new insights for clinical trial design.

### 6.2. Biomarker-Guided Clinical Trial Design

To facilitate the clinical translation of the “spatiotemporally precise network reconstruction” concept, we propose the adoption of a biomarker-enriched design, wherein patient enrollment is stratified according to pathological stage and molecular subtype:

#### 6.2.1. Liquid Biopsy as a Stratification Tool

Urinary exosomal miRNA signatures (e.g., miR-21, miR-494, miR-141) can be used to identify subgroups at “high risk of fibrosis” [146,147,151]. Studies have shown that urinary miR-494 levels increase by 60-fold within 48 h after AKI onset, with diagnostic detection occurring earlier than that of the traditional marker NGAL [146]. Plasma SASP factors (IL-6, TNF-α) can identify an “accelerated senescence” phenotype [155]. Circulating endothelial dysfunction markers (ADMA, ICAM-1, E-selectin) serve as indicators of vascular injury [154].

#### 6.2.2. Dynamic Monitoring as a Therapeutic Efficacy Indicator

Dynamic monitoring of changes in urinary NGAL, KIM-1, as well as the aforementioned miRNAs, SASP factors, and endothelial dysfunction markers during treatment can serve as pharmacodynamic indicators and early predictors of therapeutic efficacy [145,156]. During the chronic phase, circulating asymmetric dimethylarginine (ADMA) levels are associated with a more than twofold increase in all-cause mortality risk in CKD patients, serving as a surrogate endpoint for prognostic assessment [157].

### 6.3. Key Technology Platforms Required for Translation

#### 6.3.1. Liquid Biopsy Technology

Detection of exosomal miRNAs in urine and plasma has entered the exploratory phase of clinical application. Recent studies have shown that urinary exosomal miR-21-5p is highly correlated with the degree of interstitial fibrosis in kidney transplant recipients (AUC > 0.85), serving as a non-invasive monitoring tool [151]; miR-141 and miR-556-5p predict non-recovery of renal function at 90 days following AKI with superior predictive performance compared to traditional clinical indicators [147,148].

#### 6.3.2. Smart Nanocarriers

Cell-specific delivery is critical for enhancing therapeutic efficacy while reducing toxicity. Recent studies have reported tubular-targeting antioxidant nanoparticles that selectively accumulate in tubular epithelial cells in AKI models, significantly alleviating oxidative stress and inflammatory responses [158]. Self-assembled p38 peptide inhibitor nanoparticles ameliorate the AKI-to-CKD transition by inhibiting ferroptosis [159].

#### 6.3.3. AI-Driven Multi-Omics Modeling

The rapid advancement of single-cell and spatial transcriptomics has provided a data foundation for identifying key intercellular communication axes and predicting individualized intervention timing. Recent studies have leveraged integrated multi-omics analysis to identify therapeutic targets for AKI at single-cell and spatial resolution [160]. Single-nucleus RNA sequencing has mapped aberrantly active intercellular communication between repair-failed proximal tubular cells and macrophages [90]. A kidney slice culture system enabling visualization of intracellular ATP dynamics in distinct nephron segments under pathophysiological conditions provides novel insights into energy dynamics and pathogenesis in kidney disease [161]. AI algorithms can be used to integrate these high-dimensional data to construct individualized “disease trajectory prediction models,” guiding precision intervention.

### 6.4. Challenges and Future Directions

#### 6.4.1. Specificity Issues

Distinguishing the tipping point between adaptive repair and maladaptive repair represents a core current challenge. This necessitates the development of more precise dynamic markers and longitudinal monitoring strategies. For example, expression levels of p21 and p16 may reflect different phases of the cell cycle; however, their dynamic changes and relationship with clinical outcomes require validation through large-scale prospective studies [83,162].

#### 6.4.2. Safety Considerations

Senolytic interventions may affect normal tissue homeostasis and tumor suppressor functions, and their long-term safety requires validation in larger-scale clinical trials [163]. Risks such as off-target effects of CAR-T cells have not yet been fully evaluated in the context of renal fibrosis therapy [127]. The long-term biocompatibility and in vivo metabolic pathways of nanocarriers also warrant further investigation [158].

#### 6.4.3. Barriers to Clinical Translation

Current biomarkers lack standardized detection protocols and validation in large cohorts, limiting their application in multicenter clinical trials. The interpretability and generalizability of AI models require further improvement, particularly regarding their applicability across diverse etiologies and patient populations [160]. The selection of clinical trial endpoints remains controversial; the association between surrogate endpoints (e.g., eGFR slope, proteinuria) and hard endpoints (e.g., kidney failure, mortality) requires further validation.

#### 6.4.4. Ethical Considerations and Accessibility

Precision subtyping and individualized therapies may increase healthcare costs, necessitating the incorporation of health economics assessments early in the development process. Balancing “precision” with “accessibility” to ensure that novel therapies benefit broader patient populations is a critical issue that future translational research must address.

## 7. Conclusions

**Figure 4:**
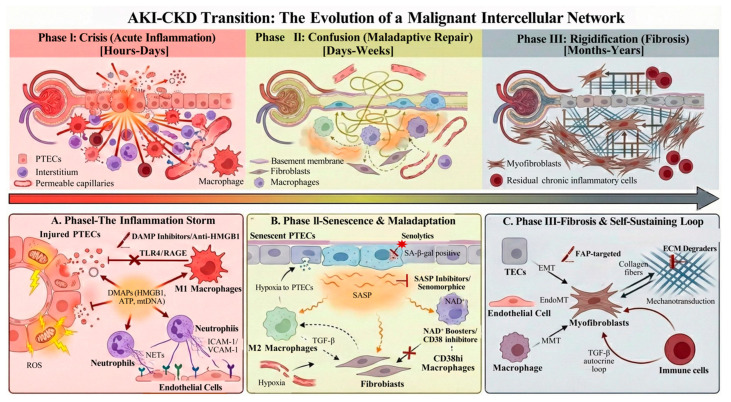
The “Malignant Social Network”: Spatiotemporal evolution of intercellular crosstalk driving the transition from AKI to CKD.

The transition from AKI to CKD is a complex process driven by multicellular interactions, from the inflammatory storm in the acute phase, to the imbalanced macrophage M1/M2 polarization and maladaptive repair of TECs in the subacute/repair phase, to the myofibroblast activation and ECM deposition in the chronic phase (Figure 4). Each stage involves dysregulation of the network among multiple cell types. The limitation of current therapeutic strategies lies in their focus on single molecular targets, which fails to disrupt the pathological network homeostasis underlying the AKI-to-CKD transition, resulting in limited clinical efficacy.

Future success in preventing and treating AKI-to-CKD progression will inevitably depend on a paradigm shift in therapeutic approach—from targeting a single molecule to remodeling the entire cellular social network. The implementation of this network remodeling strategy requires the integration of multiple technological approaches: achieving non-invasive staging of the network phase through liquid biopsy; enabling precise drug delivery to specific cells via smart nanocarriers; and predicting optimal intervention nodes and combination regimens through AI-driven multi-omics modeling. Only by constructing a spatiotemporally precise combination therapy roadmap tailored to individual patient differences can the chain of AKI-to-CKD progression be ultimately disrupted, thereby improving long-term patient outcomes.

The dynamic shift from a physiological repair process to a pathological, self-sustaining fibrotic network across three distinct phases along the disease continuum. (A) Phase I: Acute inflammation. Occurring hours to days post-injury, this phase is characterized by an “Inflammation Storm.” Injured PTECs act as crisis initiators, undergoing necroptosis or pyroptosis and releasing DAMPs (e.g., HMGB1, ATP, mtDNA). These signals activate the innate immune system, recruiting M1 macrophages and neutrophils, which release NETs via activated endothelial cells expressing ICAM-1/VCAM-1. Therapeutic opportunities (indicated by red blockage symbols) include targeting DAMP receptors (e.g., TLR4/RAGE antagonists) to dampen the initial inflammatory cascade. (B) Phase II: Maladaptive repair. Spanning days to weeks, this critical “window of opportunity” is defined by senescence and immune dysregulation. TECs with unrepairable DNA damage enter a senescent state, secreting SASP factors that act as a chronic “noise source”. Simultaneously, macrophage polarization is disrupted, characterized by persistent M1 phenotype or defective M2 repair function. Capillary rarefaction leads to hypoxia, fueling the transition. Interventions such as Senolytics (to clear senescent cells) or metabolic modulators (NAD+ boosters) are key strategies here. (C) Phase III: Fibrosis. In the chronic phase (months to years), a stable pathological ecosystem is established. Myofibroblasts, derived from multiple sources, become the dominant effectors. They deposit excessive ECM and establish self-sustaining positive feedback loops (e.g., TGF-β autocrine signaling and mechanotransduction pathways activated by matrix stiffness). Therapeutic targets include disrupting these loops via FAP-targeted therapies or ECM degradation strategies. DAMPs, damage-associated molecular patterns; ECM, extracellular matrix; EMT, epithelial–mesenchymal transition; EndoMT, endothelial-mesenchymal transition; FAP, fibroblast activation protein; HMGB1, high mobility group box 1; MMT, macrophage-to-myofibroblast transition; NETs, neutrophil extracellular traps; PTECs, proximal tubular epithelial cells; ROS, reactive oxygen species; SASP, senescence-associated secretory phenotype.

**Table 1:** Phase-Specific Intercellular Crosstalk in the AKI-to-CKD Transition.

Phase	Cell Type	Core Mediator and Key Signaling	Therapeutic Strategy
Acute Phase (hours–days)	Tubular Cells ↔ Immune Cells	DAMPs (HMGB1, ATP, mtDNA)/DAMP receptors (RAGE, TLR9, P2X7R)/ cytokines (IL-1β, TNF-α, IL-6) and chemokines (CCL2, CXCL1, CXCL2) [8,29]; ANP/CXCL1 and MPO/NETs [34].	Interventions that inhibit NETs formation [35,42]. Neutralizing the DAMP molecule HMGB1 with specific antibodies or blocking its receptor [17,56,57]. Inhibition of NF-κB (BAY 270 or 6-gingerol) [58,59].
	Endothelial Cells ↔ Immune Cells	ROS and TNF-α, IL-1β/ICAM-1, VCAM-1/LFA-1, VLA-4 [37,38,39] or CXCL8, CCL5 or thromboxane A2, angiotensin II [40,41]; NETs [42].	
	Tubular Cells ↔ Fibroblasts	TGF-β1, CTGF [44,45]; PKM2/HGF [47].	
	Immune Cells ↔ Immune Cells	Lactation of HMGB1/mtDNA/NETs [50]; MPO [52].	
	Tubular Cells ↔ Endothelial Cells	DAMPs/ROS or ET-1 [53,55].	
Subacute/Repair Phase (days–weeks)	Senescent Tubular Cells ↔ Macrophages	non-coding RNAs/TGF-β [91]; NAD [92]; Vav1/Rac2/NF-κB [93]; IFN-β [94].	MSC-EVs have been shown to promote the polarization of CX3CR1^+^ macrophages toward an M2 phenotype via the TXNIP- IKKα/NF-κBpathway [110]. HUMSC-EVs with senolytics (dasatinib and quercetin) yields superior renoprotective effects following AKI [111]. Blocking the lactate transporter MCT4, suppresses the activation of pro-inflammatory endothelial cells and thereby inhibits AKI progression [106]. Antagonism of the endothelin A receptor improves both macrovascular and microvascular function [112,113].
	Macrophages → Fibroblasts	IGF [69]; Vtn/integrin αvβ5 and Src kinase [95]; CXCL4 [96]; HIF-1α/A2BAR [98].	
	Tubular Cells ↔ Endothelial Cells	VEGF-A [99]; TNF-α [100].	
	Tubular Cells ↔ Fibroblasts	SASP [101]; MAPK/ERK/EGR1/FGF2 [102,103]; lactate/HGF [105]; PHD [106].	
	Endothelial Cells ↔ Fibroblasts	TGF-β [108]; Sema-7A/SASP [109].	
Chronic/Fibrotic Phase (months–years)	Fibroblasts ↔ Immune Cells	IRF7/CTSS [125]; Dectin-1/Syk/NF-κB/CCL2-CCR2 and TGF-β/Smad [126]; PDGFRβ [127]; IL-1β [128].	Genetic deletion or pharmacological inhibition of Dectin-1 with laminarin significantly attenuates renal fibrosis [126]. CAR-T cells engineered to specifically recognize platelet-derived growth factor receptor β (PDGFRβ) monoterpene glycoside paeoniflorin has been shown to attenuate fibroblast activation and ECM deposition by blocking the paracrine signaling of endothelial cell-derived TGF-β1 [127]. verteporfin (VP) mitigates renal inflammation and ECM deposition in UUO mice and reduces activation and proliferation of NRK-49F fibroblasts by modulating the phosphorylation of Smad2 and Smad3 [141]. The Smad3 inhibitor SIS3 alleviates fibrosis and inflammation by suppressing the TGF-β/Smad3 pathway and subsequent myofibroblast activation [142]. STAT6 inhibitor AS1517499 protects renal function by inhibiting fibroblast activation, reducing M2 macrophage polarization, and decreasing the production of ECM [143]. melatonin shows potential in enhancing cell-based therapy by improving mitochondrial function via the PrP(C)-PINK1 pathway in CKD-derived MSCs.
	Fibroblasts ↔ Tubular Cells	PCMT1/TGFBR2/TGF-β1/SMAD [130]; YAP/E2F2/FGF2 [129]; CXCR4/β-catenin/SASP [131].	
	Fibroblasts ↔ Endothelial Cells	IGFBP5/NLRP3 [133]; TGF-β1 [134].	
	Tubular Cells ↔ Immune Cells	Integrin αvβ6/IL-34 [135]; miR-155/SASP [136].	
	ECM Signaling	PSPC1 and Neat1/integrin β1/YAP/TGF-β1 [138]; Yap/Taz/TGF-β1 [139]; αvβ6 and αvβ3/FAK/ERK [140].	

## Data Availability

The original contributions presented in this study are included in the article. Further inquiries can be directed to the corresponding author.

## Abbreviations

AKI	Acute kidney injury
CKD	chronic kidney disease
SASP	senescence-associated secretory phenotype
ECM	extracellular matrix
DAMPs	damage-associated molecular patterns
ROS	reactive oxygen species
PTCs	Proximal tubular epithelial cells
HMGB1	high mobility group box 1
ATP	adenosine triphosphate
HSPs	heat shock proteins
mtROS	mitochondrial ROS
mtDNA	mitochondrial DNA
RIPK	receptor-interacting protein kinase
MLKL	mixed lineage kinase domain-like protein
GSDMD	gasdermin D
GSDME	gasdermin E
MPO	myeloperoxidase
NETs	neutrophil extracellular traps
ANP	atrial natriuretic peptide
ICAM-1	intercellular adhesion molecule-1
VCAM-1	vascular cell adhesion molecule-1
TGF-β1	transforming growth factor-β1
CTGF	connective tissue growth factor
TNF-α	tumor necrosis factor-α
IL-1β	interleukin-1β
TECs	tubular epithelial cells
IGF	insulin-like growth factor
snRNA-seq	Single-nucleus RNA sequencing
MMT	macrophage-to-myofibroblast transition
FGF2	fibroblast growth factor 2
EndMT	endothelial-to-mesenchymal transition
MSC	mesenchymal stem cell
FMT	fibroblast-to-myofibroblast transition
EMT	epithelial–mesenchymal transition
PMT	pericyte-to-myofibroblast transition
α-SMA	α-smooth muscle actin
HIFs	hypoxia-inducible factors
CAR-T	chimeric antigen receptor T
PDGFRβ	platelet-derived growth factor receptor β
YAP	yes-associated protein
